# The effect of using fresh farmyard manure (animal manure) on the severity of *Fusarium verticilioides* in soil, root, stem, and kernels as well as lodging and borer incidence of maize plants

**DOI:** 10.3389/fpls.2022.998440

**Published:** 2023-01-25

**Authors:** Samar S. A. Elsayed, Mohamed D. Sehsah, Moufida A. Oueslati, Omar M. Ibrahim, Salem Hamden, Nermien H. Seddek, Heba I. Abo-Elmagd, Dalal Hussien M. Alkhalifah, Mohamed S. Sheteiwy, Hamada AbdElgawad, Mohamed T. El-Saadony, Amira M. El-Tahan

**Affiliations:** ^1^ Maize and Sugar Crops Disease Research Department, Plant Pathology Research Institution, Agricultural Research Center, Giza, Egypt; ^2^ Plant Production Department, Arid Lands Cultivation Research Institute, The City of Scientific Research and Technological Applications, SRTA-City, Alexandria, Egypt; ^3^ Deanship of Preparatory Year and Supporting Studies and The Department of Respiratory Care, College of Applied Medical Sciences in al Jubail, Imam Abdulrahman Bin Faisal University, Dammam, Saudi Arabia; ^4^ Department of Agric. Botany (Plant Pathology), Faculty of Agriculture, Kafrelsheikh University, Kafrelsheikh, Egypt; ^5^ Department of Respiratory Care, College of Applied Medical Sciences-Jubail 4030 (CAMSJ), Imam Abdulrahman Bin Faisal University, Al Jubail, Saudi Arabia; ^6^ Department of Basic Sciences, Deanship of Preparatory Year and Supporting Studies, Imam Abdulrahman Bin Faisal University, Dammam, Saudi Arabia; ^7^ Department of Biology, College of Science, Princess Nourah bint Abdulrahman University, Riyadh, Saudi Arabia; ^8^ Department of Agronomy, Faculty of Agriculture, Mansoura University, Mansoura, Egypt; ^9^ Department of Botany and Microbiology, Faculty of Science, Beni-Suef University, Beni Suef, Egypt; ^10^ Department of Agricultural Microbiology, Faculty of Agriculture, Zagazig University, Zagazig, Egypt

**Keywords:** *Fusarium verticilioides*, farmyard, organic manure, maize, herbicides, pathogen

## Abstract

*Fusarium verticillioides*, an important maize pathogen, produce fumonisins, causes stalk rot and consequentially reduce crop growth and yield. Therefore, herein we aimed to evaluate the potential use of two farmyard soil organic manures, i.e., fresh (5-6 days old) and stored (5-6 months old) organic manure, to manage *F. verticillioides* infections as well as borer incidence and lodging in maize plants. After 30, 60, and 90 days of sowing, samples of soil, roots, and stems were collected to isolate *F. verticillioides*. Moreover, we estimated ear and kernel rot induced by *F. verticillioides* at the final harvest. Fresh organic manure treatment increased infection rates of *F. verticillioides* in soil, roots, stem and kernels compared to the control treatment. In contrast, stored organic manure plots treatments decrease *F. verticillioides* frequency. At 90 days after sowing, stored organic manure suppressed the survival of *F. verticillioides*, which reduced the *F. verticillioides* incidence percent. These results were similar to the effect of herbicides-and insecticide-treated plots demonstrated, which show a significant decrease in *F. verticillioides* incidence rates. Mycological analysis on symptomless kernels revealed a higher % of pathogen infection in opened husks variety (Balady) than closed husks variety (SC10). Compared with stored organic manure, the stem borer incidence and lodging percentage were the highest in fresh organic manure plots. Finally, these results demonstrated that storing organic manure within five to six months as farmyard manure led to high-temperature centigrade within organic manure, thereby destroying spores of *F. verticillioides*, whereas fresh organic manure did not.

## Introduction

*Fusarium verticillioides* (Sacc.) Nirenberg (syn. F. *moniliforme* J. *Sheld.*) is one of the most common causal agents of diseases in maize (Zea mays L). (Blacutt et al., 2018) indicated that F. *verticillioides* was the predominant pathogen of maize stalk rot. For instance, Fusarium stalk reduces maize yield by 10% and 30–50% in severely affected areas (Li et al., 2010). Similarly, (Feng et al., 2022) demonstrated that Fusarium species attack the stem base of maize plants, causing stalk rot – usually during August and September. A relationship has been found between the aetiology of the *Fusarium* that produces stalk rot and ear rot. (Lanubile et al., 2021) demonstrated the correlation between stalk rot caused by *Fusarium* spp. and the lodging of 35 maize cultivars after physiological maturity (PM). Applications of herbicide and manure, fungicidal treatment of seeds, manual planting, and sprinkler irrigation lead to decreased disease and good productivity (Naseri et al., 2016; Naseri and Hemmati, 2017; Adhikary et al., 2014). The occurrence of stalk rot accelerated the decomposition of soluble sugar, which accelerated the decrease of stalk strength and significantly increased the risk of stalk lodging (Xue et al., 2021). The estimated reduction in grain weight by 5-20%, whereas the loss due to Fusarium stalk rot has been reported as 38% in total yield (AICRP, 2014). It reduces the grain yield and alters the quality of the seeds. Moreover, mycotoxins of *F. verticillioides* can accumulate, threatening the health of humans and animals (Marasas, 2001; Rheeder et al., 2002; Shim et al., 2003). Therefore, it is essential to develop a mechanism to effectively exclude the pathogen from the maize plants’ rhizosphere and prevent its transmission from preceding seasons (Wilke et al., 2007; Alakonya et al., 2008).

To this end, organic amendments are applied as an innovative approach to reducing the survival and infection of maize plants by *F. moniliforme* (Alakonya et al., 2003).In this context, using organic amendments might be viable in managing *F. moniliforme* in areas where the pathogen is prevalent. Consistently, Ugo De Corato (2020) showed that plant diseases caused by soil-borne pathogens had been recognized as a critical factor worldwide for plant health and productivity overall in intensive cropping systems characterized by low organic matter content and frequent soil tillage. Under organic amendments, the infection by *Fusarium* stalk rot was estimated to reduce the grain weight by 5-20%. In contrast, the estimated loss due to *Fusarium* stalk rot has been reported as 38% in total yield (Bellini et al., 2023). The animal manure is rich in organic matter content, total biological activity and actinomycetes populations, fluorescent pseudomonads and fungi demonstrated by (Abd El-Mageed et al., 2022; Abd El-Aty et al., 2022a; Abdou et al., 2022; Abo Sen et al., 2022; Ahmed et al., 2022a; Ahmed et al., 2022b).

Alakonya et al. (2008) also reported that at 60 days after silking in the corn crop, the percent recovery of *F. verticillioides* was reduced to even zero under organic manure treatments. Mycological analysis on symptomless kernels revealed higher recovery of *F. verticilliodes* grown in control plots than amended treatments, indicating the amendment’s ability to manage root infections of *F. verticilliodes.* Ringer et al. (2013) reported that animal manure composts inhibited damping-off induced by *Pythium* and *Rhizoctonia* even more effectively than disease conducive controls. Shafique et al. (2016) showed that organic soil amendments stimulate antagonistic activities of microorganisms to soil-borne diseases and decomposition of organic matter in the soil, which resulted in bioactive antifungal or nematicidal compounds accumulation in the soil. Soils treated with a high level of organic matter reduced the *Fusarium* rot root index, and improved yields occurred (Naseri, 2014; El-Saadony et al., 2021a; Abd El-Aty et al., 2022b; El-Ashry et al., 2022; El-Marzoky et al., 2022; El-Okkiah et al., 2022a).

On the other hand, revealed that the application of herbicides reduced the population of all the tested soil-borne microorganisms during this study. It may lead to significant changes in the populations of microorganisms and their activities, thereby influencing the microbial ecological balance in the soil. According to Naseri and Hamadani (2017), the herbicide application to soil causes transient impacts on microbial population growth, mainly when applied at the recommended field application rate. Therefore, herbicides demonstrate stimulative or inhibiting effects on microbial growth depending on the chemicals (type and concentration), microbial species, and environmental conditions (Zain et al., 2013; Carles et al., 2018). Likewise, Bezuglova et al. (2019); Gao et al. (2020), and Jie et al. (2021) found that the effects of herbicides on the soil microbial environment varied based on herbicide species and soil depths. For instance, compared to atrazine and chloroacetamide, the three herbicides alachlor, acetochlor, and propisochlor have a greater tendency to inhibit the growth of microbial populations (Yadav et al., 2021).

Toxicity of herbicides can impact soil biota and human health; therefore, herein we aimed to evaluate the potential use of two farmyard soil organic manures, i.e., fresh (5-6 days old) and stored (5-6 months old) manures to manage *F. verticillioides* infections as well as borer incidence and lodging in maize plants. These treatments were used as innovative approaches to replace herbicides, killing many soil-borne pathogens and consequently causing plant diseases. Moreover, specific responses of different organs of maize cultivars, i.e. closed husks and opened husks cultivars at different growth stages, were evaluated.

## Materials and methods

### Soil, root, and stalk sample collection

Soil and root sample collection was done at 30 days, 60 days and 90 days after sowing. At every stage, a randomly selected plant from each treatment replicates was uprooted, and adventitious roots and stalks were randomly selected from every plant uprooted per plot. In addition, approximately 20 g of rhizosphere soil of uprooted plants was taken. Each of these samples was separately collected in labeled plastic collection bags. Then taken to the laboratory and stored at 4°C until they were used for account *F. verticillioides* frequency. The account proceeded within a week following sample collection (Alakonya et al., 2008).

### Recovery of *F. verticillioides* from soil, roots, and stalks

Soil samples from the three replicates 30, 60 and 90 after sowing each treatment were each mixed thoroughly, and 1g of soil was weighed for analysis. The number of colonies per gram of soil was determined by dissolving 1g of soil in 9 ml of sterile distilled water in a vial. The vial was vortexed before pipetting 1ml into another vial containing 9 ml of distilled water; this was repeated serially five times, followed by aseptically pipetting 1ml from the three last dilutions and dispensing into a separate petri dish with PDA medium. A petri dish with countable colonies provided the colonies for sub-culturing. For the roots, samples from each treatment at 30, 60 and 90 days of sowing were washed with water, dried in sterile filter paper, and then cut into 2 mm pieces. They were then placed into a separate petri dish with PDA medium (6 pieces for each petri dish). Twelve root pieces of 2 mm length per treatment/replicate were cultured 30, 60 and 90 days after sowing. The percent recovery of *F. verticillioides* was then calculated. While for stalks, the samples were taken from nodes numbers 1,2 and 3 above the crown, which was cut into cubes for about 1×1×1 cm. in a petri dish with PDA medium (6 cubes for each petri dish) at each treatment and each date. All were incubated at 25 centigrade for 5 days. The percent recovery of *F. verticillioides* was then calculated (Alakonya et al., 2008).

### Field maize harvesting and data collection: Two rows

Per plot samples were harvested after physiological maturity (120 days of sowing). Information collected in the field included yield per two rows at each treatment, percentage lodging and borer incidence.

### Recovery of *F. verticillioides* from kernels

Samples were taken after 100, 110 and 120 days of sowing. Three ears from each treatment at each date were randomly taken and dried on laboratory benches and room temperature until moisture content fell between 13 -15%. Moisture content below 13% prevents the growth of saprophytic fungi and hence maintains the sample’s integrity. Briefly, the kernels were dipped in 1% sodium hypochlorite for two minutes before rinsing twice in sterile distilled water and drying between sterile filter papers. Ten seeds were then plated on PDA media in three replicates and incubated at 25°C for five days. The colonies of *F. verticillioides* isolates were estimated (Alakonya et al., 2008; Riley, 2021).

### Preparation of the animal manure

The animal’s organic manure was collected by farmers from farm animal barns daily. Then moved and stored in the farmyard and left until it was used in the field. This study used two kinds of animal organic manures: The first was fresh animal organic manure (stored for 5-6 days before using), and the second was stored animals organic manure (stored for about 5-6 months before using).

### Field experiment

This experiment was conducted at Sakha Agric. Res. Station Farm, during 2019 and repeated during 2020 growing seasons. Split-split plot design with three replicates was used; the main plots were two maize cultivars (single cross (s.c) 10 and balady), the sub-plots were the tested dates (30, 60 and 90 days of sowing), and the sub-sub plots were six treatments as follows: 1- soil treatment with fresh animal manure and herbicide; 2- soil treatment with fresh animal manure and no herbicide; 3-soil treatment with stored animal manure and herbicide; 4- soil treatment with stored animal manure and no herbicide; 5-soil untreated with animal manure and treated with herbicide, and 6- soil untreated each of animal manure and herbicide (control). Each experiment unit (plot) consisted of four rows, 5m long and 80 cm width, and each row contained 20 hills, 25cm apart. Animal manure soil was treated after sowing and irrigation for about 1kg/row (ton per Feddan). The soil treatment by herbicide (Harness for about 1litre per Feddan) was also sprayed after sowing and before irrigation; the trade name, rate of application and active ingredient were shown in **Table 1**. All agricultural practices were applied as recommended time. The first and the second rows in each plot were used for taking the soil, roots, stalks and kernels samples in each treatment at each tested date. At the same time, the other two rows in each plot were used to estimate the lodging percent, insect incidence and yield per two rows at harvest.

**Table 1:** Trade name, rate of application, and active ingredient of harness herbicide as a spray on the soil surface after planting and before irrigation.

Trade name	Rate of application	Active ingredient
Harness EC84%	1L/Feddan	Acetachrol

## Results and dissection

To deal with *F. verticilliodes* infection and its mycotoxins, many approaches have been applied in agriculture; however, they are usually based on the use of herbicides that are reported to be acutely and chronically unsafe for humans and animals and to have adverse effects on the ecosystems (Li et al., 2019; Yadav et al., 2021). Alternative approaches to respecting the ecosystems and human and animal health have been developed. Notably the application of organic amendments as a strategy for controlling diseases caused by soil-borne pathogens. In this context, organic amendments have been reported to reduce pathogen viability and diffusion in soils and plant organs (Thi Nguyen et al., 2019). However, these effects depend on plant age, organs, and cultivars. Thus, this study was carried out to evaluate the antifungal potential of two organic amendments against F. verticillioides infection of different maize organs and at different growth stages.

In conclusion, different levels of F. *verticillioides* contamination were detected in maize plants grown under field conditions depending on their growth stage.

### Stored organic manure reduced *F. verticilliodes* frequency in the rhizosphere of SC10 and Balady maize cultivars

**Figure 1:**
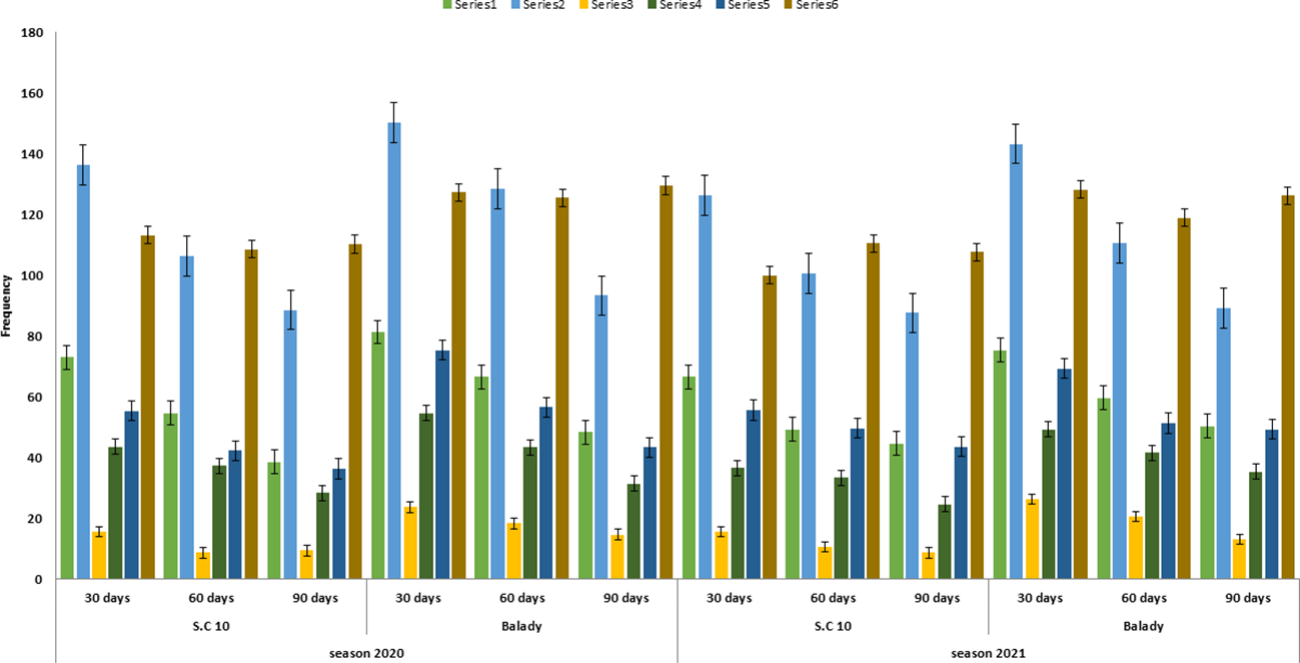
*F. verticillioides* frequency from soil under different soil treatments at different times after sowing during the 2020 and 2021 growing seasons. Treatment1= soil was treated with fresh animal manure and herbicide. Treatment 2= soil was treated with fresh animal manure and untreated with herbicide. Treatment 3= soil treated with 5-6 months old animal manure and herbicide. Treatment 4= soil was treated by 5-6 months old animal manure and untreated with herbicide. Treatment 5= soil was untreated with any animal manure and treated with herbicide. Treatment 6=soil was Untreated either of any animal manure and/or herbicide.

Organic amendments are often used to improve soil quality as they can change the general suppressiveness of soil because of the enriched microbial activity. Interestingly, the frequency of *F. verticillioides* largely depended on the type of organic manure.*F. verticilliodes* was approximately equally present in S.C 10 and Balady maize cultivars (**Table 2** and **Figure 1**). However, it was increased in the rhizosphere of the balady cultivar. Moreover, the lowest *F. verticillioides* was recorded in understored and herbicide treatments. In this regard, it recorded 15.66, 8.66 and 9.33 in S.C 10 and 23.66, 18.33 and 14.66 in balady cultivar after 30, 60 and 90 days of sowing, respectively. At the same time, the highest *F. verticillioides* frequency was recorded with treatment soil by fresh animal manure (5-6 days old) or control treatments. They recorded 136.33, 106.33 and 88.66 in S.C 10 and 150.33, 128.66 and 93.33 in balady cultivar after 30,60 and 90 days of sowing, respectively. These results were similar during the two seasons (2022 and 2021). These results are in the same line with that reported by Shafique et al. (2016), who showed that organic soil amendments stimulate antagonistic activities of microorganisms to soil-borne diseases. Bio-fertilizers have been reported to increase the abundance of biological control in soils (Thi Nguyen et al., 2019). These biological controls prevent plant diseases and mycotoxin contamination by controlling the propagation of phytopathogens and toxin production (Naz et al., 2021).

**Table 2:** *Fusarium verticillioides* frequency from soil under different treatments at different times after sowing during the 2020 growing season.

Maize cultivars	Sc 10			Balady		
Days of sowing	30 days	60 days	90 days	30 days	60 days	90 days
T1	73.00c	54.66c	38.66c	81.33c	66.66c	48.33c
T2	136.33a	106.33b	88.66b	150.33a	128.66a	93.33b
T3	15.66f	8.66f	9.33f	23.66f	18.33f	14.66f
T4	43.66e	37.66e	28.33e	54.66e	43.66e	31.33e
T5	55.33d	42.33d	36.33d	75.33d	56.66d	43.33d
T6	113.33b	108.66a	110.33a	127.33b	125.66b	129.66a
L.S.D 0.01 L.S.D 0.05	1.615	1.125	1.548	0.774	0.608	1.912
	1.134	0.796	1.087	0.543	0.427	1.511

(T1) Fresh animal manure and herbicide treated (T2) Fresh animal manure and herbicide untreated (T3) 5-6 months old animal manure and herbicide treated (4) 5-6 months old animal manure and herbicide untreated (T5) Untreated with animal manure and herbicide treated (T6) Untreated either of animal manure and/or herbicide.

Moreover, Ringer et al. (2013) showed a positive correlation between animal manure age and its effective suppression of microorganisms. Similarly, herbicides decrease the frequency of *F. verticillioides* (**Tables 2**, **3**). These results were in line with the results of Bezuglova et al. (2019); Gao et al. (2020); Kepler et al., 2020 and Jie et al. (2021). They treated the soil with herbicides and revealed that its application reduced the population of all the tested microorganisms. The effect was increased with increasing the concentration of herbicides. Overall, the application of bio-organic fertilizer can control the *Fusarium* wilt of cucumber plants by regulating the microbial community of rhizosphere soil

**Table 3:** *Fusarium verticillioides* frequency from soil under different treatments at different times after sowing during the 2021 growing season.

Maize cultivars	Balady			Sc 10		
Days of sowing	90 days	60 days	30 days	90 days	60 days	30 days
T1	50.33c	59.66c	75.33c	44.66c	49.33c	66.66c
T2	89.33b	110.66b	143.33a	87.66b	100.66b	126.33a
T3	13.00e	20.66f	26.33f	8.66f	10.66e	15.66f
T4	35.33d	41.66e	49.33e	24.66e	33.33d	36.66e
T5	49.33c	51.33d	69.33d	43.66d	49.66c	55.66d
T6	126.33a	119.00a	128.33b	107.66a	110.66a	100.00b
L.S.D 0.01 L.S.D.0.05	7.713	5.264	4.256	1.053	5.153	3.531
	5.420	3.694	2.981	0.740	3.621	2.484

(T1) Fresh animal manure and herbicide treated (T2) Fresh animal manure and herbicide untreated (T3) 5-6 months old animal manure and herbicide treated (4) 5-6 months old animal manure and herbicide untreated (T5) Untreated with animal manure and herbicide treated (T6) Untreated either of animal manure and/or herbicide.

### Cultivar and treatment-specific response to organic manure induced *F. verticilliodes* frequency reduction in maize organs

The frequency of *F. verticillioides* largely depends on the maize growth stage, cultivar and treatment. Moreover, the biological control by organic manure amendment, a practical approach to suppress the *Fusarium* wilt through inhibiting the soil-borne pathogens, is also maize cultivar and organ-specific.

### Infection of maize root

**Figure 2:**
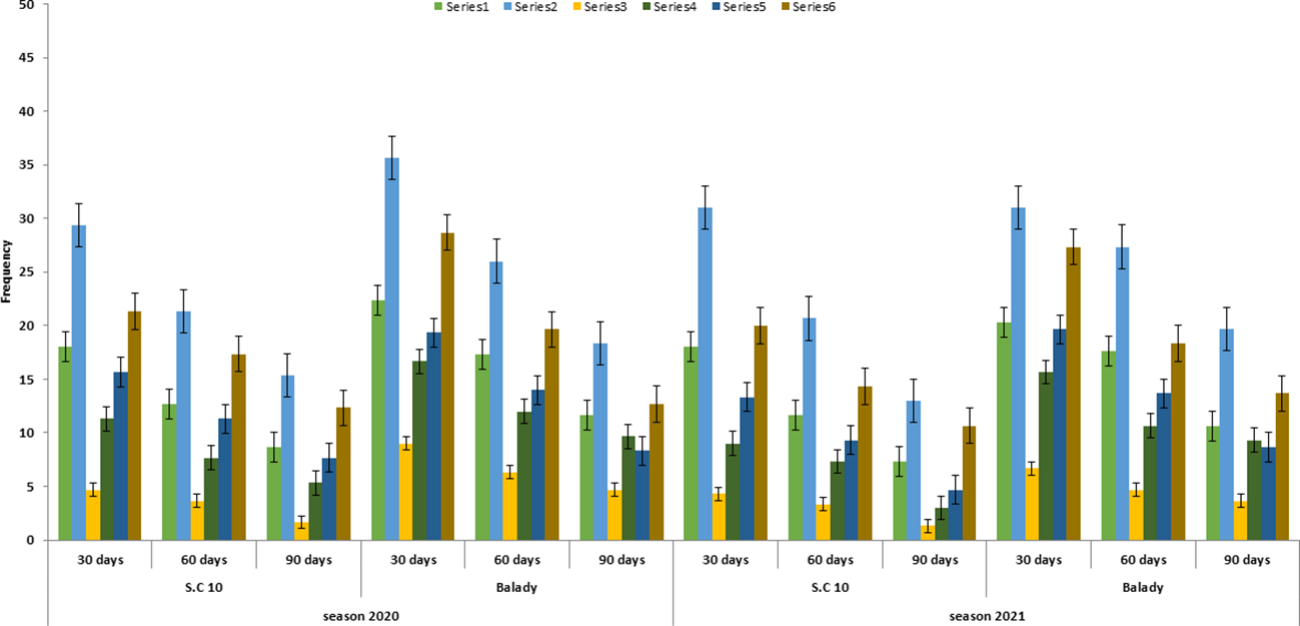
*F. verticillioides* frequency % from roots under different soil treatments at different times after the sowing date during the 2020 and 2021 growing seasons.

The pathogenesis is responsible for the formation of seedling blight and root rot, which limit seedling emergence and plant development (Faidallah et al., 2022; Hassan et al., 2021; Khairy et al., 2022; Khedr et al., 2022; Naiem et al., 2022a; Naiem et al., 2022b; Tuon et al., 2022). *F.verticilliodes* frequency in roots was highest after 30 days of sowing and gradually decreased after 60 and 90 days. (**Table 4** and **Figure 2**). This *F.verticilioides* frequency was lower under soil fertilizer stored organic manure (5-6 months storage) and herbicide treatment; this recorded 4.33, 3.33 and 1.33 in S.C 10, and 6.66, 4.66 and 3.66 in balady cultivar after 30, 60 and 90 days from sowing, respectively. While the highest *F. verticillioides* frequency in roots under fresh organic manure (5-6 days old). Consistently, these results confirmed the season of 2021 (**Table 5**). These results are in the same line as reported by Ringer et al. (2013), who added a positive correlation between animal manure age and its effective suppression of microorganisms. These results clarify the importance of soil fertilization with stored organic manure (for about five to six months). In general, the biocontrol of plant root disease can be obtained by manipulating the rhizosphere microflora by favouring beneficial microorganisms that are directly antagonistic to root pathogens (Gardiner, 2018, El-Saadony et al., 2022b, El-Sitiny et al., 2022, Elnahal et al., 2022, Fouda et al., 2022, Gadallah et al., 2022).

**Table 4:** *Fusarium verticillioides* frequency % from roots under different soil treatments at different times after sowing during the 2020 growing season.

Maize cultivars	Sc 10			Balady		
Days of sowing	30 days	60 days	90 days	30 days	60 days	90 days
T1	18.00c	12.66c	8.66c	22.33c	17.33c	11.66b
T2	29.33a	21.33a	15.33a	35.66a	26.00a	18.33a
T3	4.66f	3.66e	1.66e	9.00f	6.33e	4.66e
T4	11.33e	7.66d	5.33d	16.66e	12.00d	9.66c
T5	15.66d	11.33c	7.66c	19.33d	14.00d	8.33d
T6	21.33b	17.33b	12.33b	28.66b	19.66b	12.66b
L.S.D at 0.01	2.393	2.265	1.634	3.322	3.043	1.615
L.S.D at 0.05	1.686	1.598	1.155	2.346	2.148	1.134

(T1) Fresh animal manure and herbicide treated (T2) Fresh animal manure and herbicide untreated (T3) 5-6 months old animal manure and herbicide treated (4) 5-6 months old animal manure and herbicide untreated (T5) Untreated with animal manure and herbicide treated (T6) Untreated either of animal manure and/or herbicide.

**Table 5:** *Fusarium verticillioides* frequency % from roots under different soil treatments at different times after sowing during the 2021 growing season.

Maize cultivars	Sc 10			Balady		
Days of sowing	30 days	60 days	90 days	30 days	60 days	90 days
T1	18.00c	11.66c	7.33c	20.33c	17.66b	10.66c
T2	31.00a	20.66a	13.00a	31.00a	27.33a	19.66a
T3	4.33f	3.33f	1.33f	6.66e	4.66e	3.66e
T4	9.00e	7.33e	3.00e	15.66d	10.66d	9.33d
T5	13.33d	9.33d	4.66d	19.66c	13.66c	8.66d
T6	20.00b	14.33b	10.66b	27.33b	18.33b	13.66b
L.S.D at 0.01	1.125	0.774	2.051	1.868	1.615	1.615
L.S.D at 0.05	0.796	0.543	1.447	1.316	1.134	1.134

(T1) Fresh animal manure and herbicide treated (T2) Fresh animal manure and herbicide untreated (T3) 5-6 months old animal manure and herbicide treated (4) 5-6 months old animal manure and herbicide untreated (T5) Untreated with animal manure and herbicide treated (T6) Untreated either of animal manure and/or herbicide.

### Infection of maize stalks

**Figure 3:**
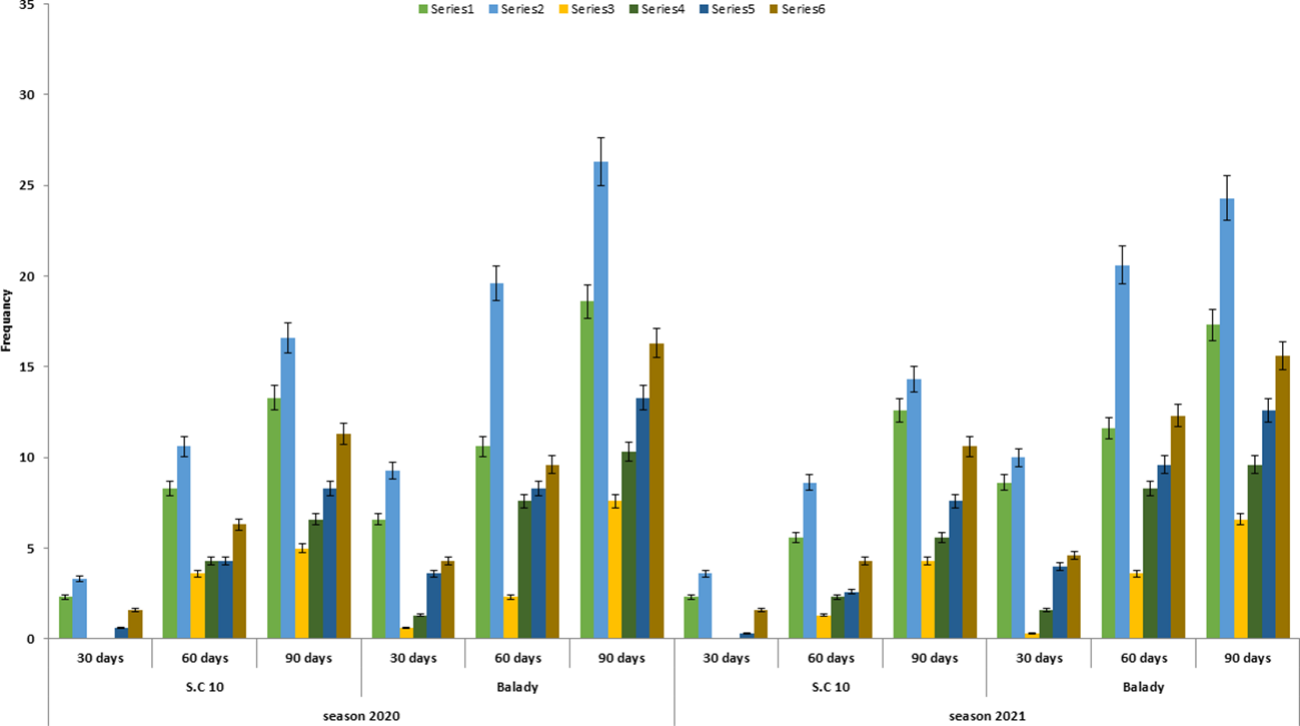
*F. verticillioides* frequency % from stalks under different tested treatments at different tested times after the sowing date during the 2020 and 2021 growing seasons.

The lowest frequency of *F. verticillioides* from maize stalks after 30 days of sowing is shown in (**Table 6** and **Figure 3**). This frequency was lower (0.00 to 3.33) in S.C 10maize cultivar. On the other hand, the highest frequency of tested pathogens was recorded after 90 days of sowing. At the treatment level hand, storage of organic manure (5-6 months old) or with herbicide induced more inhibition of *F. verticillioides* frequency in maize stalks compared to fresh organic manure (**Table 7**). *F. verticillioides* frequency was also lower in S.C 10 cultivar (0.00, 3.66 and 5.00) than in the Balady maize cultivar (0.66, 2.33 and 7.66) after 30, 60 and 90 days of sowing, respectively. These results were similar to that reported by Alakonya et al. (2008), who reported that at 60 days after silking, where recovery % of *F. verticillioides* was reduced to even zero in some treatments indicating that organic soil amendments have a mechanism of suppressing the survival of *F. verticilliodes* in the soil and hence limit its root and stalk infection ability.

**Table 6:** *Fusarium verticillioides* frequency % from stalk under different soil treatments at different times after sowing during the 2020 growing season.

Maize cultivars	Sc 10			Balady		
Days of sowing	30 days	60 days	90 days	30 days	60 days	90 days
T1	2.33b	8.33b	13.33b	6.66b	10.66b	18.66b
T2	3.33a	10.66a	16.66a	9.33a	19.66a	26.33a
T3	0.00c	3.66d	5.00f	0.66d	2.33d	7.66f
T4	0.00c	4.33d	6.66e	1.33d	7.66c	10.33e
T5	0.66c	4.33d	8.33d	3.66c	8.33c	13.33d
T6	1.66b	6.33c	11.00c	4.33c	9.66b	16.33c
L.S.D at 0.01	1.248	1.986	1.886	1.567	1.868	2.099
L.S.D at 0.05	0.877	1.39	1.325	1.105	1.316	1.479

(T1) Fresh animal manure and herbicide treated (T2) Fresh animal manure and herbicide untreated (T3) 5-6 months old animal manure and herbicide treated (4) 5-6 months old animal manure and herbicide untreated (T5) Untreated with animal manure and herbicide treated (T6) Untreated either of animal manure and/or herbicide.

**Table 7:** *Fusarium verticillioides* frequency % from stalk under different soil treatments at different times after sowing during the 2021 growing season.

Maize cultivars	Sc 10			Balady		
Days of sowing	30 days	60 days	90 days	30 days	60 days	90 days
T1	2.33b	7.66b	13.66b	8.66a	10.66b	17.33b
T2	3.66a	10.66a	15.33a	10.00a	17.66a	24.33a
T3	0.00c	3.33e	5.33f	0.33c	2.66d	6.66f
T4	0.00c	4.33de	6.66e	1.66c	7.33c	9.66e
T5	0.33c	4.66d	8.66d	4.00b	8.66c	12.66d
T6	1.66b	6.33c	11.66c	4.66b	11.33b	15.66c
L.S.D at 0.01	1.248	1.826	0.774	3.193	2.145	1.615
L.S.D at 0.05	0.877	1.283	0.543	2.244	1.508	1.134

(T1) Fresh animal manure and herbicide treated (T2) Fresh animal manure and herbicide untreated (T3) 5-6 months old animal manure and herbicide treated (4) 5-6 months old animal manure and herbicide untreated (T5) Untreated with animal manure and herbicide treated (T6) Untreated either of animal manure and/or herbicide.

### Infection of maize kernels

**Figure 4:**
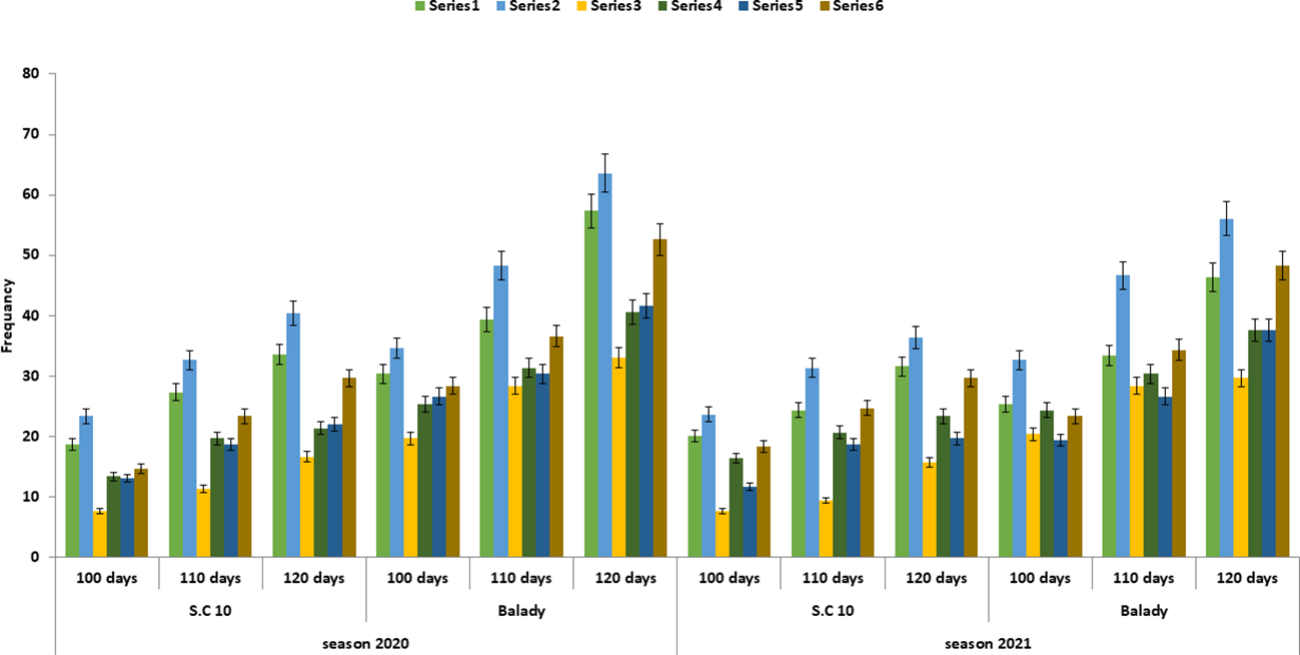
*F. verticillioides* frequency % from kernels under different tested soil treatments at different tested times after sowing during the 2020 and 2021 growing seasons.

Our data (**Table 8** and **Figure 4**) matches the lowest frequency of *F. verticillioides* in maize kernels after 100 days of sowing in all tested treatments. This frequency ranged from 7.66 to 23.33% and 19.66 to 34.66% in S.C 10 and Balady maize cultivars, respectively. At the same time, the highest frequency of tested pathogen was after 120 days of sowing, where it ranged from 16.66 to 40.33% and from 33.00 to 63.33% in S.C 10 and Balady maize cultivars, respectively. On the other hand, the lowest *F. verticillioides* frequency in maize stalks was recorded under soil treatment with storage animal manure (5-6 months old) and under treatment with herbicide at all times (**Table 9**). Here, we recorded 7.66, 11.33 and 16.66% in S.C 10 and 19.66, 28.33 and 33.00% in Balady maize cultivars after 100,110 and 120 days of sowing, respectively. In contrast, the highest *F.verticilioides* frequency was recorded under soil treatment by fresh animal manure (5-6 days old) and untreated with herbicide. In contrast, it recorded 23.33, 32.33 and 40.33% in S.C 10 and 34.66, 48.33 and 63.33% in Balady maize cultivars after 100, 110 and 120 days of sowing, respectively. These results agreed with Wilke et al. (2007) and Alakonya et al. (2008), who found that after 60 days of silking, % recovery of *F. verticillioides* was inhibited; this also indicates that organic soil amendments can suppress the survival of *F. verticilliodes* in the soil and hence limit its root infection as well as in salk, stem ear and kernels. These results were also supported by Lina et al. (2019) study, which indicated that *F. verticillioides* was the predominant pathogen of maize stalk rot. A relationship between the aetiology of the *Fusarium* that produces stalk rot and ear rot has been found. The same kind of *Fusarium* from both diseases can cross-infect. Maize stalk rot aggravates the occurrence of ear rot.

**Table 8:** *Fusarium verticillioides* frequency % from kernels under different soil treatments at different times after sowing during the 2020 growing season.

Maize cultivars	Sc 10			Balady		
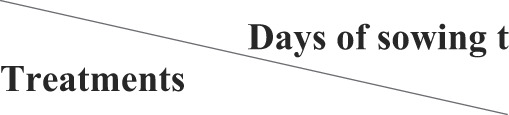	100 days	110 days	120 days	100 days	110 days	120 days
T1	18.66b	27.33b	33.33b	30.33b	39.33b	57.33b
T2	23.33a	32.33a	40.33a	34.66a	48.33a	63.33a
T3	7.66d	11.33e	16.66e	19.66d	28.33c	33.00e
T4	13.33c	19.66d	21.33d	25.33c	31.33c	40.66d
T5	13.00c	18.66d	22.00d	26.33bc	30.33c	41.66d
T6	14.66bc	23.33c	29.66c	28.33bc	36.66b	52.66c
L.S.D at 0.01	6.435	4.189	2.653	6.046	5.627	5.276
L.S.D at 0.05	4.525	2.948	1.86	4.24	3.951	3.703

(T1) Fresh animal manure and herbicide treated (T2) Fresh animal manure and herbicide untreated (T3) 5-6 months old animal manure and herbicide treated (4) 5-6 months old animal manure and herbicide untreated (T5) Untreated with animal manure and herbicide treated (T6) Untreated either of animal manure and/or herbicide.

**Table 9:** *Fusarium verticillioides* frequency % from kernels under different soil treatments at different times after sowing during the 2021 growing season.

Maize cultivars	Sc 10			Balady		
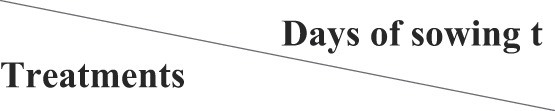	100 days	110 days	120 days	100 days	110 days	120 days
T1	20.00b	24.33b	31.66b	25.33b	33.33b	46.33b
T2	23.66a	31.33a	36.33a	32.66a	46.66a	56.00a
T3	7.66f	9.33e	15.66f	20.33d	28.33d	29.66d
T4	16.33d	20.66c	23.33d	24.33bc	30.33c	37.66c
T5	11.66e	18.66d	19.66e	19.33d	26.66e	37.66c
T6	18.33c	24.66b	29.66c	23.33c	34.33b	48.33b
L.S.D at 0.01	1.868	1.567	1.615	1.467	2.291	3.821
L.S.D at 0.05	1.316	1.105	1.134	1.036	1.617	2.694

(T1) Fresh animal manure and herbicide treated (T2) Fresh animal manure and herbicide untreated (T3) 5-6 months old animal manure and herbicide treated (4) 5-6 months old animal manure and herbicide untreated (T5) Untreated with animal manure and herbicide treated (T6) Untreated either of animal manure and/or herbicide.

### Lodging and insects incidence % are reduced by stored organic manure amendment

**Figure 5:**
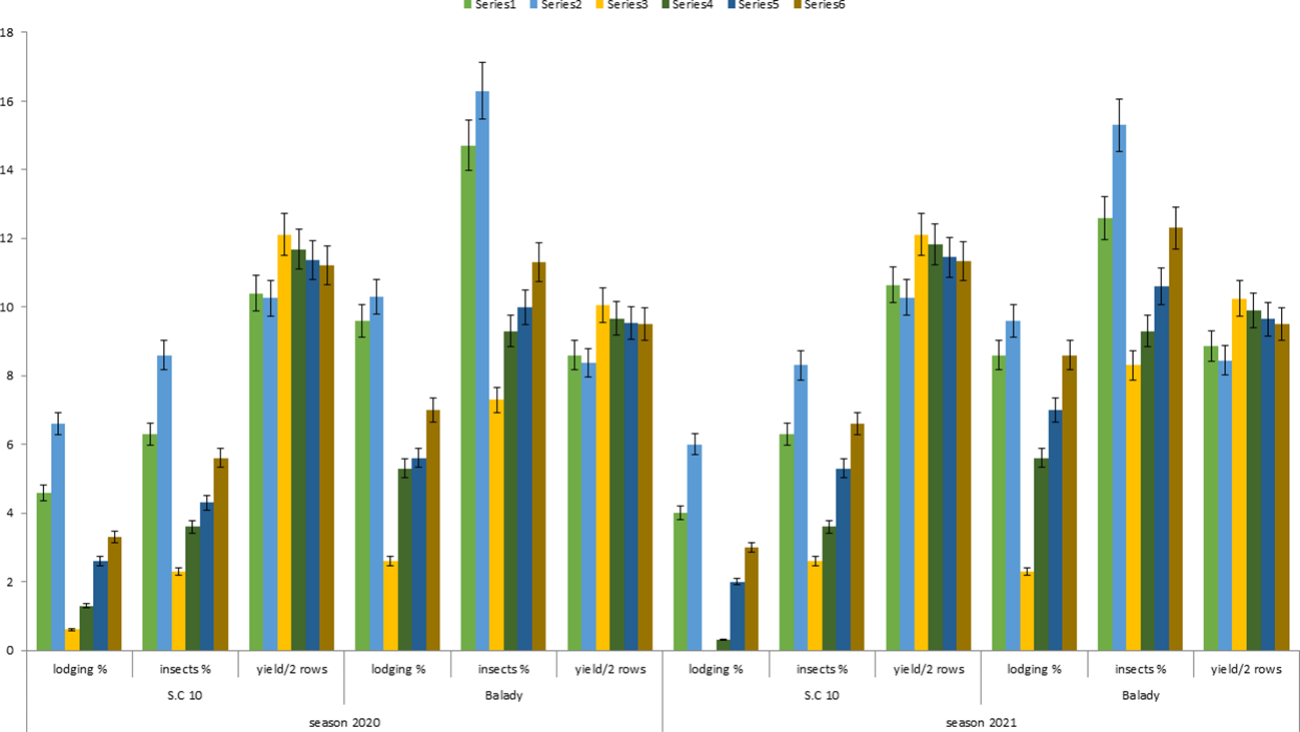
Percentage of lodging, insects incidence, and yield per two rows (kg) under the tested treatments during the 2020 and 2021 growing seasons.

Our data (**Table 10** and **Figure 5**) obtained during the 2020 growing season and at the final harvest indicated that the insects infection % and lodging % were increased in the case of fresh animal manure (5-6 days old). This treatment increased insects infection % and lodging % by 8,66 and 6.66%, respectively, as well as the lowest yield per two rows (10.260 g.) in S.C 10. In comparison, it recorded 16.33 and 10.33%, respectively, as well as the lowest yield per two rows (8.370 g.) in the Balady maize cultivar. On the other hand, the insect infection % and lodging % were decreased in the case of treatment with storage animal manure (5-6 months old) and herbicide, since they recorded 2.33 and 0.66%, respectively. The highest yield per two rows (12.116 g.) in S.C 10, while it recorded 7.33 and 2.66%, respectively, as well as the highest yield per two rows (10.050 g.) in the Balady maize cultivar. Here, during the 2021 growing season, the same trend as those reported in the first year (**Table 11**). These results align with those reported by Echezona (2007), who found that cultural practices that increase the amount of insect pressure, such as corn densities and planting geometries, can also increase the amount of lodging in the corn crop. These results also agreed with (Lanubile et al., 2021), who found a relation between stalk rot caused by *Fusarium* spp. and lodging in maize. They also significantly and positively correlated with the soluble sugar and stalk lodging percentage. The occurrence of stalk rot accelerated the decomposition of soluble sugar, which accelerated the decrease of stalk strength and greatly increased the risk of stalk lodging.

**Table 10:** Percentage of lodging, insect incidence and yield per two rows (kg) under the tested treatments during the 2020 growing season.

Maize	SC10			Balady		
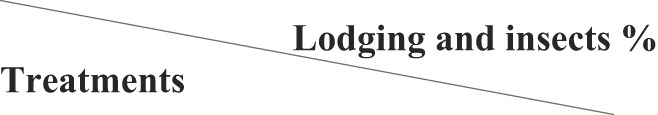	Lodging %	Insects %	Yield/two rows	Lodging %	Insects %	Yield/two rows
T1	4.66b	6.33b	10.403d	9.66a	14.66a	8.603c
T2	6.66a	8.66a	10.260d	10.33a	16.33a	8.370d
T3	0.66d	2.33d	12.116a	2.66c	7.33d	10.050a
T4	1.33d	3.66c	11.683b	5.33b	9.33c	9.666b
T5	2.66c	4.33c	11.366c	5.66b	10.00bc	9.526b
T6	3.33c	5.66b	11.216c	7.00b	11.33b	9.500b
L.S.D at 0.01	1.548	1.634	0.224	2.61	2.497	0.305
L.S.D at 0.05	1.087	1.155	0.152	1.832	1.754	0.211

(T1) Fresh animal manure and herbicide treated (T2) Fresh animal manure and herbicide untreated (T3) 5-6 months old animal manure and herbicide treated (4) 5-6 months old animal manure and herbicide untreated (T5) Untreated with animal manure and herbicide treated (T6) Untreated either of animal manure and/or herbicide.

**Table 11:** Percentage of lodging and insect incidence under the tested treatments during 2021 growing seasons.

Maize	SC10			Balady		
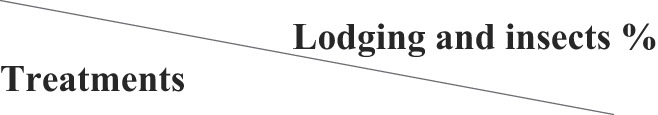	Lodging %	Insects %	Yield/ rows	Lodging %	Insects %	Yield/ rows
T1	4.00b	6.33b	10.65d	8.66b	12.66b	8.850d
T2	6.00a	8.33a	10.28e	9.66a	15.33a	8.450e
T3	0.00d	2.66c	12.166a	2.33e	8.33d	10.250a
T4	0.33d	3.66c	11.83b	5.66d	9.33cd	9.900b
T5	2.00c	5.33b	11.45c	7.00c	10.66c	9.650bc
T6	3.00bc	6.66b	11.35c	8.66b	12.33b	9.516c
L.S.D at 0.01	1.467	1.947	0.361	1.412	1.986	0.455
L.S.D at 0.05	1.036	1.364	0.25	0.993	1.39	0.32

(T1) Fresh animal manure and herbicide treated (T2) Fresh animal manure and herbicide untreated (T3) 5-6 months old animal manure and herbicide treated (4) 5-6 months old animal manure and herbicide untreated (T5) Untreated with animal manure and herbicide treated (T6) Untreated either of animal manure and/or herbicide.

### Correlation analysis

**Figure 6:**
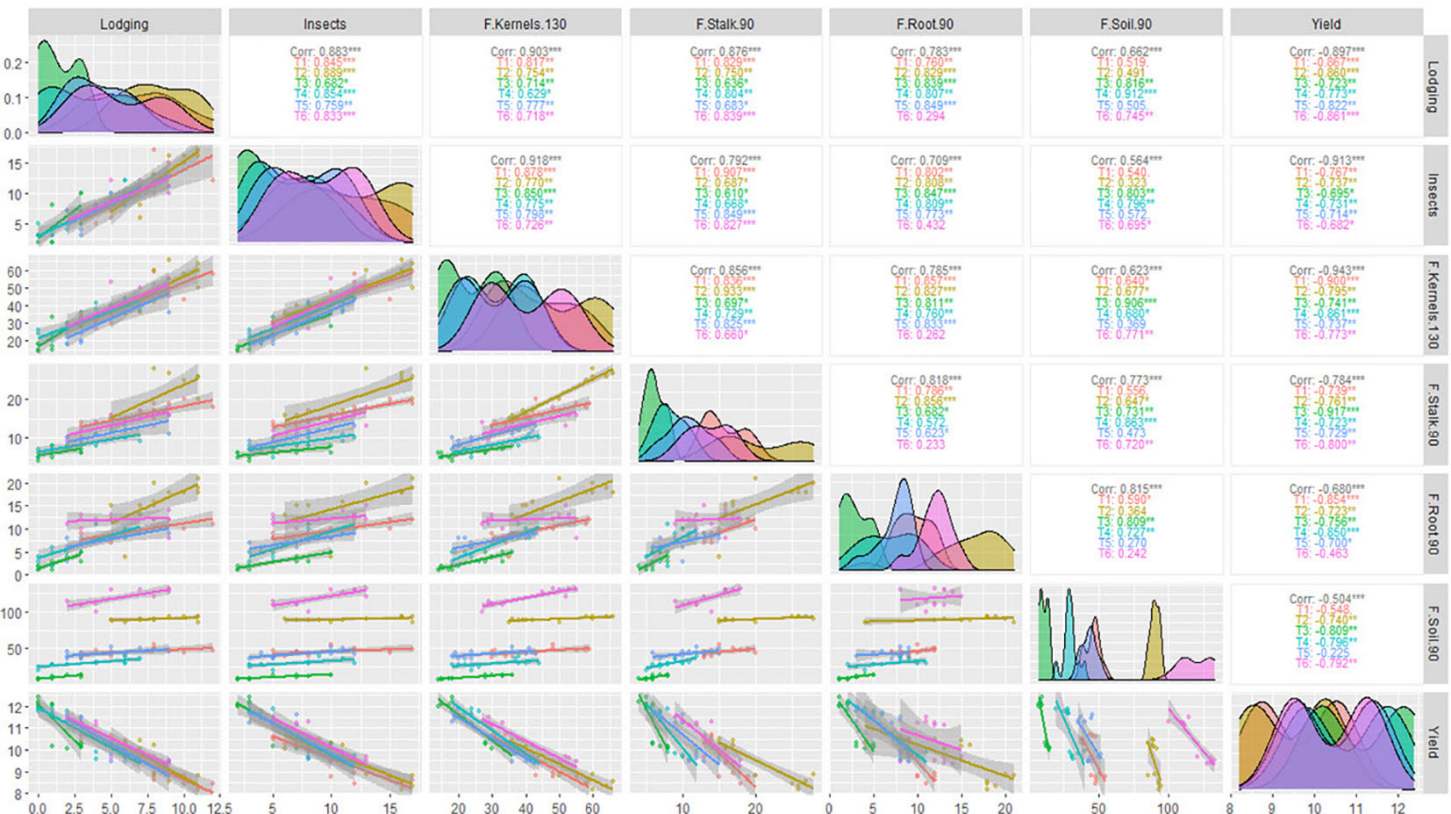
Spearman correlation matrix among the studied traits.

**Figure 6** shows the correlation matrix among the studied traits for the six treatments. The yield was negatively and significantly correlated with all the studied traits under all the treatments except for fusarium frequency in root at 90 days in T6 treatment and fusarium frequency in the soil at 90 days in T5. There was a positive and significant correlation between the percentage of lodging, insects, and fusarium frequency in soil and maize organs (root and kernels). In addition, the figure depicts the density plots of the six treatments using kernel density estimation to estimate the probability density function (PDF). Each density plot shows the density or relative probability of the values. The area under the curve represents the distribution shape of the values of each treatment, showing whether or not a distribution has one peak (unimodal), two peaks (bimodal), or multiple peaks (multimodal) of occurring values frequency (Karev and Artem, 2019). The values on the Y axis represent the density or relative probability of the corresponding values, while the values on the X axis represent each trait’s values. A higher concentration or higher density of the values of each trait for a given treatment is indicated by the peak in each density plot. Simultaneously, the tails indicate a lower density or concentration of each trait for a given treatment, which means a lower relative probability. Most of the density plots have two peaks indicating the two cultivars, except for *Fusarium* in the soil at 90 days; the distribution of traits in all six treatments appeared extensive. At 90 days, there is a lower variation in the frequency of Fusarium in the soil, as demonstrated by the results. The lower triangle part of the figure shows the linear relationship among the studied traits over the six treatments with confidence intervals. The relationship reflects the linearity of the association among the studied traits, particularly between yield and the remainder of the studied traits.

### Heatmap cluster analysis confirmed cultivar-specific responses

**Figure 7:**
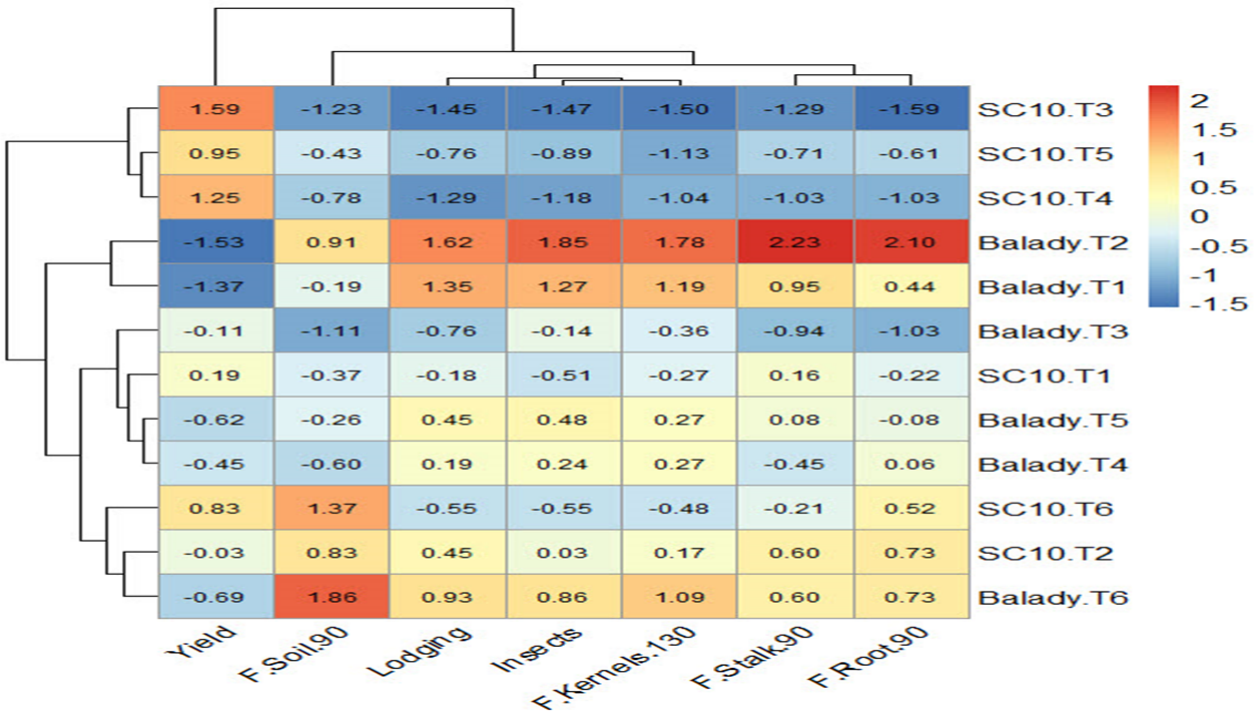
Heatmap showing the relationship between the studied traits and the treatments.

Heatmap was produced using the (*pheatmap*) package and the function (*pheatmap*) in (R Core Team, 2021). Heatmap cluster analysis (**Figure 7**) shows a relationship between treatments and the studied traits based on scaled (standardized) data presented in the color scale. Red color cells in the heatmap represent high values of the traits, while blue color cells represent low values of the traits. Before constructing the heatmap cluster analysis, the data were rescaled or standardized by subtracting the mean of each trait from each value and dividing the result by the standard deviation of that trait. Rescaling or standardization process is used to make comparison feasible because the studied traits were measured in different units. The results reveal that cultivar SC10 under T3 treatment was the highest yield (red color), which was associated with the lower values of the other traits (blue values). Cultivar Balady recorded the lowest yield under T1 and T2 (blue color), which was associated with higher values of the other traits (red color), except for fusarium frequency in the soil at 90 days which was lower in the association.

### Artificial neural networks

**Figure 8:**
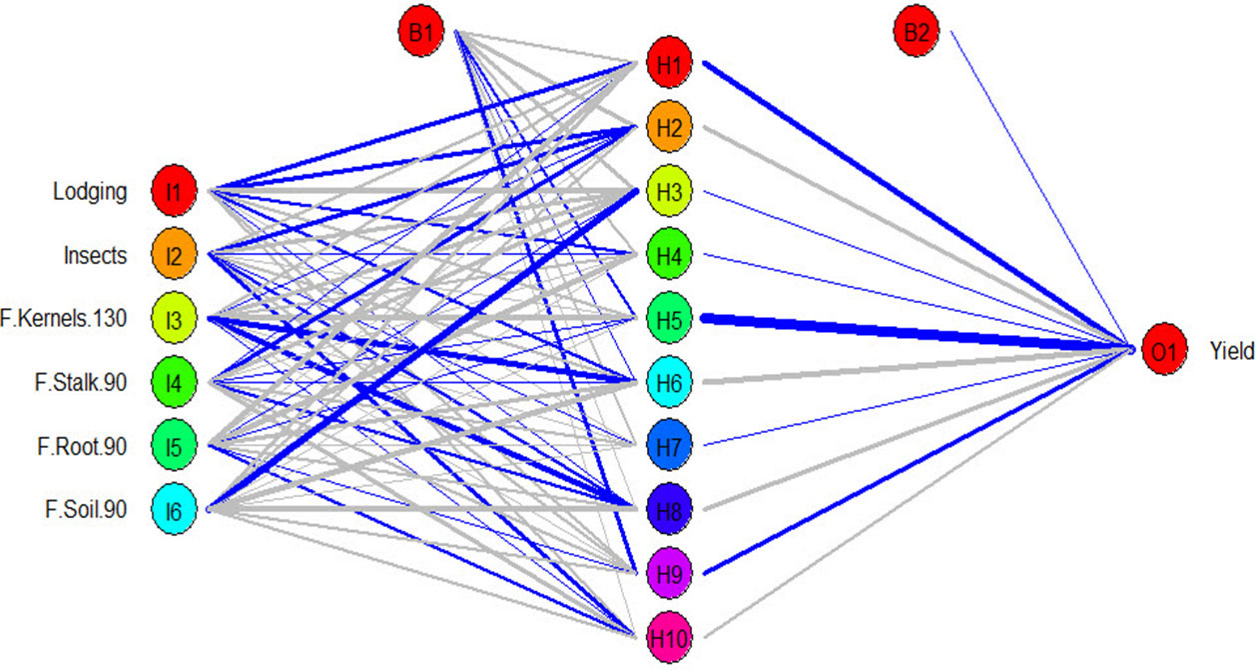
The structure of the used artificial neural network in the present study.

**Figure 9:**
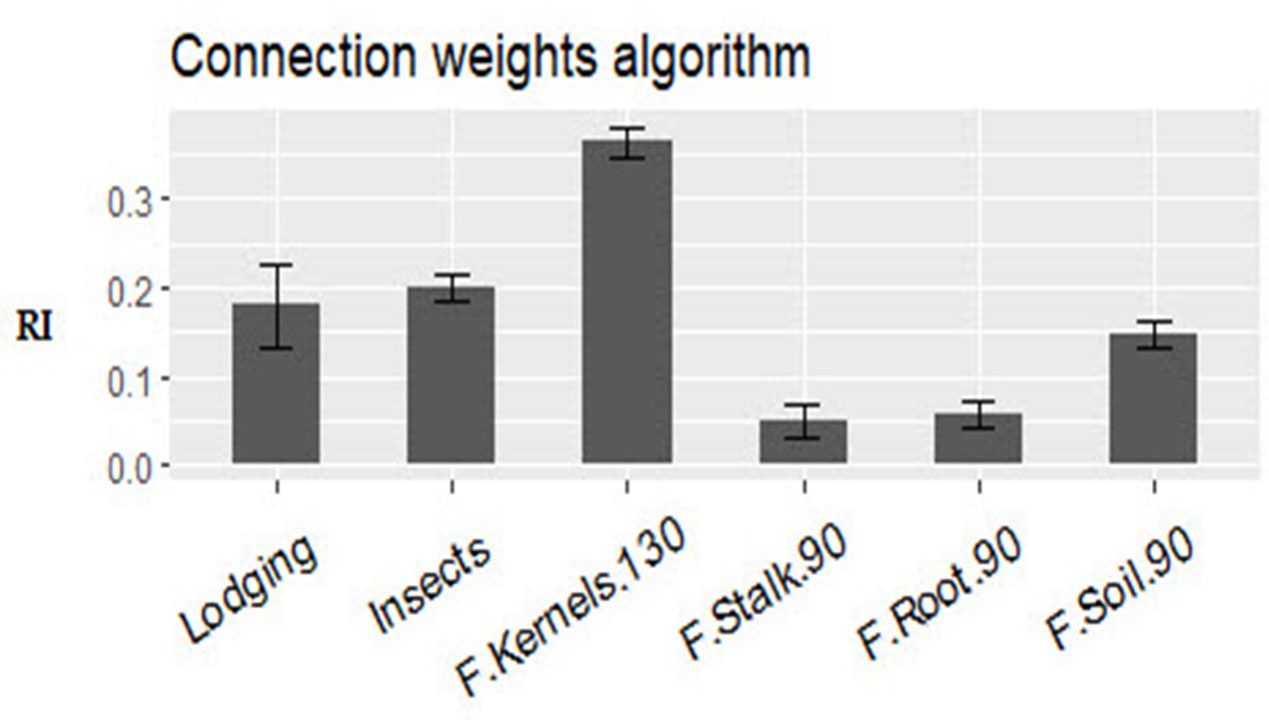
The relative importance of the studied traits to the yield.

Artificial neural networks are robust models interconnected by numerous and simple processing units known as neurons with a structure parallel to the biological neurons in human brains (Zhongheng, 2016). Neurons are arranged in sets called layers, such as the input layer, hidden layer, and output layer (**Figure 8**). The neurons in one layer are connected to the neurons in the next layer but not those in the same layer. The strength of the connection weights among two neurons between the layers is used to estimate the relative importance of the inputs to the output as a percent. The present study used a three-layer feed-forward multilayer perceptron neural network (MLP) using a backpropagation algorithm. We revealed that *Fusarium* frequency in kernels at 130 days was an essential trait to the yield (36%); insects came second in importance (20%), while lodging was third (19%) (**Figure 9**). *Fusarium* frequency in the soil at 90 days had 15% importance. Lastly, *Fusarium* frequency in stalk and root at 90 days was the least essential trait (5% for each).

## Conclusion

The integrated approach of *F. verticillioides* could involve the use of resistant plant hybrids, the use of hybrids that have closed husks, the use of grains treated by soil-borne fungicides, the use of some herbicides which kill some soil-borne pathogens, good post-harvest date and handling practices, and use of storage organic manure (for about five to six weeks); this is because increasing the temperature centigrade within stored organic manure could kill many eggs and spores of insects and the tested pathogen, respectively, and caution never uses fresh organic manure.

## Data availability statement

The original contributions presented in the study are included in the article/supplementary material. Further inquiries can be directed to the corresponding author.

## Author contributions

Conceptualization, SE, MDS, OI, ME-S, and AE-T; methodology, SE, MDS, OI, ME-S, and AE-T; formal analysis, SE, MDS, OI, ME-S, and AE-T; investigation, SE, MDS, OI, ME-S, and AE-T; data curation, SE, MDS, OI, ME-S, and AE-T; writing—original draft preparation, SE, MDS, OI, ME-S, and AE-T; writing—review and editing SE, MDS, OI, ME-S, and AE-T. All authors have read and agreed to the published version of the manuscript.
